# Sex Differences in Soft Tissue Sarcoma: Incidence, Clinicopathological Profile, Survival, and Costs

**DOI:** 10.1089/jwh.2023.0019

**Published:** 2023-10-31

**Authors:** Alessandra Buja, Massimo Rugge, Saveria Tropea, Claudia Cozzolino, Carlo Maria Formaro, Giulia Grotto, Manuel Zorzi, Antonella Vecchiato, Paolo Del Fiore, Antonella Brunello, Marta Sbaraglia, Eliana Ferroni, Carlo Riccardo Rossi, Angelo Paolo Dei Tos, Simone Mocellin

**Affiliations:** ^1^ Laboratory of Healthcare Services and Health Promotion Evaluation, Hygiene and Public Health Unit, Department of Cardiac, Thoracic and Vascular Sciences, and Public Health, University of Padua, Padua, Italy.; ^2^ Pathology and Cytopathology Unit, Department of Medicine (DIMED), University of Padua, Padua, Italy.; ^3^ Veneto Tumor Registry, Azienda Zero, Padua, Italy.; ^4^ Soft-Tissue, Peritoneum, and Melanoma Surgical Oncology Unit and Veneto Institute of Oncology (IOV)–IRCCS, Padua, Italy.; ^5^ Medical Oncology Unit 1, Veneto Institute of Oncology (IOV)–IRCCS, Padua, Italy.; ^6^ Department of Pathology, Azienda Ospedale Università Padova, Padua, Italy.; ^7^ Servizio Epidemiologico Regionale, Azienda Zero, Padua, Italy.; ^8^ Department of Surgery, Oncology, and Gastroenterology (DISCOG), University of Padua, Padua, Italy.

**Keywords:** soft tissue sarcoma, gender difference, incidence, real-world cost, survival

## Abstract

**Background:**

There are evident sex differences in the incidence of and mortality rates for several tumors. Soft tissue sarcomas (STSs) account for no more than 1% of all malignancies in adults. This study aimed to provide a comprehensive overview of the sex differences in the epidemiology of STSs and the related costs.

**Methods:**

This retrospective population-based study draws on epidemiological data regarding cases of STS collected by the cancer registry of the Italian Veneto region for the years 1990–2018. A joinpoint regression analysis was performed to identify significant changes in the trends of the standardized incidence rates in males and females. Bivariate and survival analyses were conducted to assess differences in clinicopathological characteristics and short-term mortality by sex. Direct health care costs incurred over 2 years after a diagnosis of STS were calculated, stratified by sex.

**Results:**

The incidence rates of STS at any age were higher for males; only among males the incidence rates showed a tendency to slightly increase. No significant sex differences came to light in short-term mortality or clinicopathological profile, except for the cancer site. Health care costs in the 2 years after a diagnosis of STS were not sex related.

**Conclusion:**

The STS incidence was found to be higher for males and showed a rising trend over the last three decades only for males. These findings could result from the occupational exposure to environmental mutagens mainly involving men. Sex did not affect the survival or the clinicopathological STS profile.

## Introduction

Soft tissue sarcomas (STSs) are mesenchymal malignant tumors that account for no more than 1% of all cases of cancer.^1,2^ The STS spectrum includes different neoplastic histotypes^3^ classified basically by their (putative) cell lineage of differentiation as adipocytic, fibroblastic/myofibroblastic, fibrohistiocytic, smooth muscle, pericytic, skeletal muscle, vascular or chondro-osseous neoplasms; nerve sheath tumors; tumors of uncertain differentiation; or undifferentiated/unclassified sarcoma.^4^ The most common histological subtypes are leiomyosarcomas (14%) and liposarcomas (10%); some of STSs (16%) lack any consistent lineage of differentiation (so-called unclassified sarcomas).^5,6^ The age-standardized incidence of STS (excluding gastrointestinal stromal tumors [GISTs]) in Europe is reportedly 4.20–4.71 per 100,000 population.^7,8^ As for the related mortality in Western countries, the 5-year relative survival rate for STS in adults is around 59%–60%,^9,10^ but ranges widely (from 15% to 80%) for different histotypes, tumor stages at presentation, and primary site.^11^

Sex differences in the STS incidence and mortality are apparent across a wide age range, and many different cancer types.^12^ Most STSs with clear sex differences affect males more than females.^1,13^ In addition to developing STSs more often, males are also more likely to die of this disease.^1,14,15^ Despite the evidence supporting such differences, the role of sex has not been consistently investigated. On the incidence of STS, a population-based epidemiological analysis conducted in Europe in the years 1996–2015 found a slightly higher STS age-standardized incidence rate per 100,000 person-years for males.^6^ Previous research also showed that the frequency of histological subtypes of STS (other than GISTs) varied between the two sexes: fibrosarcomas (14%) and liposarcomas (12%) were the most common in males; complex mixed and stromal neoplasms (22%), nonuterine leiomyosarcomas (10%), and fibrosarcomas (9%) in females.^16^

Few studies have addressed the costs of STS using real-world data,^17,18^ and—to the best of our knowledge—none has compared the costs of health care for STS in men as opposed to women. A comprehensive assessment of sex differences among cases of STS is still lacking. The aim of the present study was therefore to obtain a detailed picture of the sex differences in the incidence of STS, its clinicopathological profile, prognosis, and related costs, using data from a population-based regional cancer registry.

## Methods

### Context

The Italian public health care system (National Health Service [NHS]) is managed regionally. It provides universal coverage, largely free of charge, at the point of delivery, the costs being covered primarily by general taxation.^19^ Its policies are grounded on the fundamental values of universality, free access, freedom of choice, pluralism in provision, and equity.

In 2015, the Veneto Oncology Network—Rete Oncologica Veneta (ROV) published a document detailing the procedures for the clinical management of patients with STS based on the current national and international literature.^20,21^ It contains guidelines on all aspects from diagnosis to end-of-life care, and a set of indicators for assessing the consistency between these recommendations and real-world clinical practice.^22–24^

### Clinical data

This retrospective population-based study draws on epidemiological data regarding cases of STS (excluding GIST, Kaposi's sarcoma, Ewing's sarcoma, uterine and visceral sarcomas) in patients of all ages collected by Veneto's regional cancer registry–Registro Tumori del Veneto (RTV) from 1990 to 2018.

The RTV includes a high-resolution database recording several anatomopathological characteristics of adult (>19 years old) incident cases of STS anywhere in the Veneto (covering the whole population of 4.9 million) in 2017 and 2018. The following variables were considered for the present study: age and sex; tumor site (classified as limbs, retroperitoneum, trunk, head, or neck); primary tumor diameter (mm), histology grade, depth, combined clinicopathological TNM stage at diagnosis (I, II, III, or IV, according to the American Joint Committee on Cancer 7th edition); and mitotic count (per high-power field). Although the histological subtype (International classification of diseases for oncology - 3rd edition code) was also available, for this analysis, cases of sarcoma were grouped into major families by cell differentiation (according to the 2020 WHO Classification of Soft Tissue Tumors^25^) as liposarcoma; fibroblastic/myofibroblastic (*e.g.*, fibromyxoid sarcomas or dermatofibrosarcoma protuberans); undifferentiated or not otherwise specified sarcomas; leiomyosarcoma; vascular (*e.g.*, angiosarcoma); and other (rare morphologies).

These incident cases were linked with the mortality registry to record vital status (as at the end of follow-up on December 31, 2021).

### Cost analysis

The cost analysis was conducted from a health system perspective. Data on visits to outpatient clinics, specialist services, drug prescriptions, hospital or hospice admissions, treatments at the emergency department, and the use of medical devices were obtained from the regional administrative subject-level databases, as done in a previous study.^17^ The costs of any diagnostic and therapeutic (surgical or other) interventions were based on the reimbursement rates established by the Veneto Regional Authority. For the cost assessment, we specifically considered the following sources:

- the outpatient database, which contains information on all medical procedures (specialist visits, laboratory and radiological tests, radiotherapy and chemotherapy sessions, *etc.*) delivered at outpatient facilities under Italian NHS funding, valued at the rate stated in the Tariff Nomenclature for Outpatient Services, a detailed formulary of medical procedures for outpatients^26^;
- the hospital admission database, which includes the diagnosis-related group for each admission, valued at the rate indicated in the Tariff Nomenclature for Inpatient Services, a formulary covering all hospital activities, including day hospital admissions^27^;
- the regional databases of outpatient drug prescriptions and in-hospital drug consumption, which records the costs of all medical therapies (including their dosage);
- the emergency department admission database, which records the cost of each admission, as the sum of all medical procedures undertaken;
- the medical device database, which lists the costs of all medical devices reimbursed by the NHS (tailored devices, disposable devices, and medical aids for rare diseases)^28^;
- the hospice database, which records admissions and the length of each stay.

Each patient was linked to all administrative data *via* a unique, anonymous identification code. All costs sustained over 2 years after a case of STS was diagnosed were included in the present analysis. The survival-weighted real-world cost statistics per patient were calculated and stratified by sex. Only individuals alive at the start of each year were considered for the computation and estimates were weighted adjusting by the observed person time. All costs are in euros.

### Statistical analysis

The 1990–2018 temporal trends in the incidence of STS in the Veneto Region (standardized for the European population in 2013) were calculated by sex. Then a joinpoint regression analysis was performed to identify significant changes in the yearly trends of the standardized incidence rates for males and females.^29^ For each trend identified, the annual percent change (APC) was also calculated by fitting a regression line to the natural logarithm of the rates, using the calendar year as a regression variable. The age-standardized incidence of STS in 2017–2018 was calculated by stratifying by sex. The European population was considered the standard reference and was extracted using the Eurostat CensusHub2 tool.^30^ Descriptive statistics are given as frequencies and percentages, while continuous numerical variables are summarized using means, medians, standard deviations (SD), and minimum–maximum intervals.

A bivariate analysis was run to compare demographics and clinical characteristics of STS between males and females. The Mann–Whitney U test and the chi-squared test (or Fisher's exact test for frequencies smaller than 5) were used to assess the statistical differences in means and proportions, respectively. Differences in short-term mortality by sex were first tested using Kaplan–Meier curves and the log-rank test. Then a multivariable Cox regression model (adjusting for age, tumor site, and TNM stage) was performed. Differences by sex in the likelihood of accessing the surgical procedure was also tested using logistic regression (adjusting for age, tumor site, and TNM stage).

Results were deemed statistically significant at the *p* < 0.05 level. Data analyses were conducted using R software.^31^ Data visualizations were obtained using the Python Plotly library.^32^

### Ethics

To ensure confidentiality and anonymity, the Veneto Regional Authority removes all direct identifiers, which are always replaced by a code number in all data sets to retain the opportunity to link data from different administrative databases. The data analysis was performed using anonymous aggregated data with no chance of individuals being identified. Ethical approval for the study was obtained from the Veneto Oncological Institute's Ethics Committee (No. 0001218/22).

### Informed consent statements

The study complied with the Declaration of Helsinki, and with Resolution No. 9/2016 of the Italian Guarantor for the Protection of Personal Data, which also confirmed the allowability of processing personal data for medical, biomedical, and epidemiological research, and that data concerning people's health can be used in aggregate form in scientific studies. In the present case, there was no need to obtain patients' written consent as per Resolution No. 9/2016 of the Italian Guarantor for the Protection of Personal Data.

## Results

**FIG. 1.**
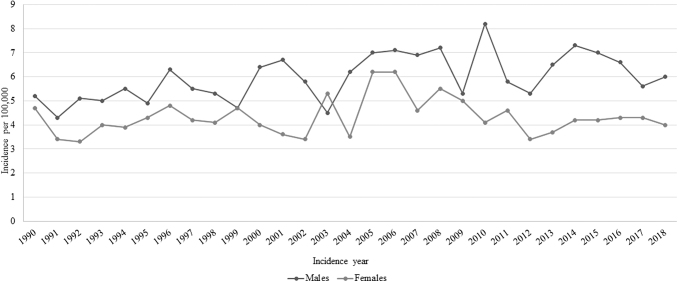
Trends in the soft tissue sarcoma incidence by sex (years 1990–2018).

Figure 1 shows the trends in the standardized incidence rates of STS by sex from 1990 to 2018. The corresponding APC estimates are shown in Table 1. In the time interval investigated, the incidence rates of STS at any age were always higher for males than for females (except 2004). While the curve remained flat for women, there was a slightly rising trend for men: the APCs for the overall period 1990–2018 were 0.9 (*p*-value <0.001) for males and 0 (*p*-value >0.05) for females.

**Table 1.** Soft Tissue Sarcoma Incidence Annual Percent Change and 95% Confidence Intervals for the Years 1990–2018 in the Population of the Veneto Region, by Sex

Sex	Period	APC (95% CI)
Males	1990–2018	0.9^**^ (0.2–1.5)
Females	1990–2018	0 (−0.8 to 0.8)
Total	1990–2006	2.0^**^ (0.7–3.4)
	2006–2018	−1.2 (−2.7 to 0.2)

Statistically significant: ^**^p-value <0.001.

APC, annual percent change; CI, confidence interval.

**FIG. 2.**
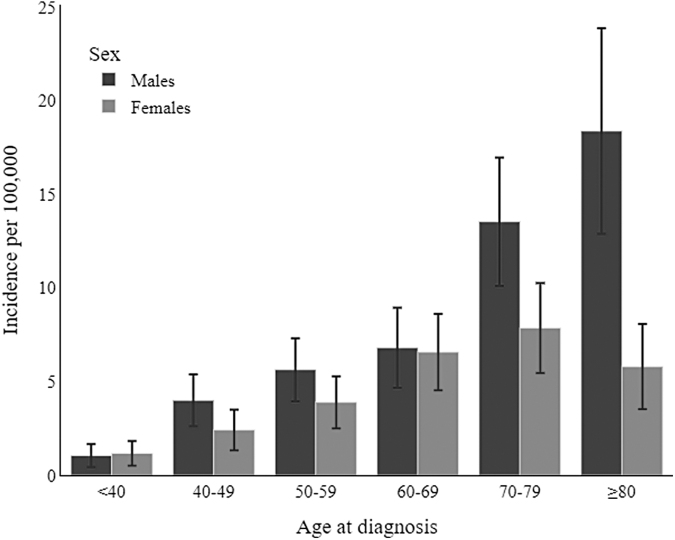
2017–2018 Incidence of soft tissue sarcomas in the Veneto Region, by age and sex.

The 2017–2018 cohort included 404 adult cases of STS: 173 (42.82%) were female and 231 (57.18%) were male. The mean age of patients at diagnosis was 64.41 (SD ±15.53) years. The crude and age-standardized STS incidence rates for 2017–2018 are shown in Table 2. The rates were higher in males than in females (with crude rates of 8.21 and 5.55, respectively, and age-standardized rates of 5.46 and 3.98, respectively, per 100,000). The incidence rates by age group were again higher in males than in females, approaching a statistically significant difference for the ≥80-year olds (18.37, 95% confidence interval [CI] = 12.88, 23.86 per 100,000 for males vs. 5.80, 95% CI = 3.53, 8.07 for females) (Fig. 2).

**Table 2.** 2017–2018 Incidence Rate of Soft Tissue Sarcomas per 100,000 (95% Confidence Interval)

	All STS cases		Females		Males	
Crude rate	6.807	(6.144–7.471)	5.545	(4.719–6.371)	8.207	(7.148–9.265)
Rate by age						
<40	1.105	(0.653–1.556)	1.165	(0.506–1.824)	1.046	(0.428–1.664)
40–49	3.209	(2.329–4.09)	2.411	(1.327–3.495)	3.995	(2.611–5.379)
50–59	4.759	(3.667–5.85)	3.892	(2.5–5.285)	5.633	(3.95–7.317)
60–69	6.687	(5.212–8.162)	6.575	(4.537–8.612)	6.806	(4.67–8.942)
70–79	10.466	(8.425–12.507)	7.859	(5.453–10.264)	13.534	(10.11–16.958)
≥80	10.223	(7.793–12.652)	5.799	(3.526–8.072)	18.373	(12.882–23.863)
Age-standardized (European standard population)						
	4.703	(3.477–5.929)	3.979	(2.369–5.588)	5.458	(3.617–7.3)

STSs, soft tissue sarcomas.

**FIG. 3.**
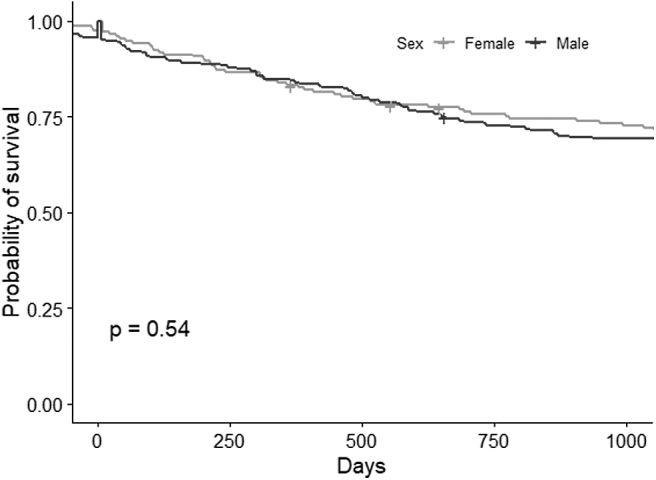
Kaplan–Meier curves by sex, log-rank *p*-value.

The STS patients' pathology did not differ significantly between the sexes, except for tumor site (*p*-value = 0.026). STSs involving the retroperitoneum were more frequent in females (28.32% vs. 18.61%), whereas males were more likely to have STS in the limbs (44.16% vs. 38.15% in females), or head and neck (13.42% vs. 6.94% in females) (Table 3). The Kaplan–Meier curves (Fig. 3) and Cox regression analysis revealed no significant differences in survival by sex. A hazard ratio of 1.12 (*p*-value = 0.544) and 1.20 (*p*-value = 0.391) was estimated for males (reference females) on univariate analysis and with the multivariable model (adjusting for age, TNM stage, and tumor location).

**Table 3.** Characteristics of Soft Tissue Sarcomas at Diagnosis by Sex

	All STS cases		Females		Males		*p*-Value
	Value	% (*N* = 404)	Value	% (*N* = 173)	Value	% (*N* = 231)	
Age (at diagnosis), in years							0.359
Mean	64.41		63.5		65.1		
Median	66		66		67		
SD	15.53		15.3		15.7		
Min–Max	20–95		20–94		20–95		
Year of diagnosis							0.666
2017	190	47.03	84	48.55	106	45.89	
2018	214	52.97	89	51.45	125	54.11	
Primary site							0.026^*^
Limbs	168	41.58	66	38.15	102	44.16	
Retroperitoneum	92	22.77	49	28.32	43	18.61	
Trunk	91	22.52	42	24.28	49	21.21	
Head and neck	43	10.64	12	6.94	31	13.42	
Unknown	10	2.48	4	2.31	6	2.60	
Size							0.316
≤100 mm	191	47.28	87	50.29	104	45.02	
101–150	27	6.68	14	8.09	13	5.63	
>150 mm	55	13.61	24	13.87	31	13.42	
Unknown	131	32.43	48	27.75	83	35.93	
Tumor depth							1.000
Superficial	150	37.13	64	36.99	86	37.23	
Deep	229	56.68	97	56.07	132	57.14	
Unknown	25	6.19	12	6.94	13	5.63	
Cell differentiation							0.523
Liposarcoma	102	25.25	36	20.81	66	28.57	
Fibroblastic/myofibroblastic	92	22.77	44	25.43	48	20.78	
Undifferentiated or NOS	91	22.52	39	22.54	52	22.51	
Leiomyosarcoma	71	17.57	34	19.65	37	16.02	
Vascular	18	4.46	7	4.05	11	4.76	
Other	67	16.58	12	6.94	17	7.36	
TNM stage (AJCC 7th edition)							0.056
I	105	25.99	56	32.37	49	21.21	
II	110	27.23	48	27.75	62	26.84	
III	102	25.25	37	21.39	65	28.14	
IV	48	11.88	21	12.14	37	16.02	
Unknown	29	7.18	11	6.36	18	7.79	
Grade							0.079
G1	90	22.28	49	28.32	41	17.75	
G2	74	18.32	31	17.92	43	18.61	
G3	187	46.29	71	41.04	116	50.22	
GX	49	12.13	21	12.14	28	12.12	
Unknown	4	0.99	1	0.58	3	1.30	
Mitoses per 10 HPF							0.391
0–9	67	16.58	32	18.50	35	15.15	
10–19	35	8.66	15	8.67	20	8.66	
≥19	46	11.39	16	9.25	30	12.99	
Unknown	256	63.37	110	63.58	146	63.20	
Three-year survival							0.571
Yes	280	69.31	123	71.10	157	67.97	
No	124	30.69	50	28.90	74	32.03	

Statistically significant: ^*^p-value < 0.05.

AJCC, American Joint Committee on Cancer; HPF, high-power field; NOS, not otherwise specified; SD, standard deviations.

**FIG. 4.**
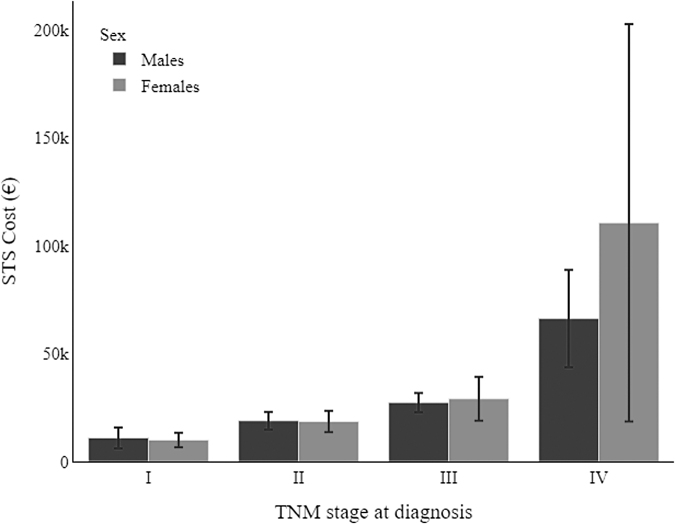
Survival-weighted mean costs of health care for soft tissue sarcomas at 2 years after diagnosis, by sex and TNM stage.

Figure 4 and Table 4 show the health care costs incurred in the 2 years after STS was diagnosed. The survival-weighted mean and median costs did not show any significant differences by sex. Moreover, testing the likelihood of accessing the surgical procedure, the univariate model gave an odds ratio (OR) for being resected of 0.62 (reference female, *p* = 0.100), while the model adjusted for age, TNM stage, and site gave an OR = 0.36 (*p* = 0.05), a value approaching statistical significance.

**Table 4.** Survival-Weighted Mean Costs (in €) at 2 Years After Diagnosis and 95% Confidence Interval, by TNM Stage and Sex

	Females				Males			
TNM stage	Mean	95% CI	Median	[Min–max]	Mean	95% CI	Median	[Min–max]
I	9945	(6629–13,261)	4899	[0–72,787]	10,907	(6041–15,773)	5883	[55–95,367]
II	18,506	(13,515–23,496)	14,566	[443–82,944]	18,861	(14,701–23,020)	14,140	[0–78,289]
III	29,051	(18,898–39,203)	17,152	[1154–156,043]	27,293	(22,811–31,775)	21,133	[1093–72,995]
IV	110,514	(18,520–202,508)	26,552	[6252–241,356]	66,176	(43,662–88,689)	24,674	[894–196,024]

## Discussion

The present study found that the overall incidence of STS increased from 1990 to 2018, with higher incidence rates in males. The trend in the incidence of STS among women remained flat over the years, while it rose lightly for males. Our analysis of the available STSs' clinicopathological profiles did not reveal any significant sex-related difference, with the only exception of tumor site. The mortality risk for patients with STS was much the same for both sexes, and so were the survival-weighted mean and median costs incurred to manage the disease.

Returning to the sex differences in the incidence of STS, the higher incident rates in males emerging in the present study are consistent with the literature.^33–36^ A previous study conducted in three European regions (Rhone-Alps, Aquitaine, and Veneto) on a sample of ∼26 million person-years identified 525 cases of STS in males versus 443 in females (crude rates: 3.98 vs. 3.19 per 100,000, respectively).^36^

As for the STS incidence rates continuing to rise in recent years only for males, in the present study sample, a possible explanation could relate to occupational exposure to potential mutagens in jobs mainly performed by men. In fact, previous studies on the association between occupational exposures and sarcoma found a higher incidence among gardeners, railroad workers, farmers, farm managers, and workers in the pulp and paper industry, on construction sites, at chemical plants, meatpacking and woodworking installations, and nuclear facilities.^37,38^ Several environmental genotoxic agents—such as vinyl chloride, dioxin, and chlorophenol—have been investigated for their potential promoting role in the STS pathogenesis.^39,40^

Exposure to pesticides is one of the most extensively examined factors in epidemiological studies, with considerable attention being paid to the potentially harmful effects of phenoxy herbicides and chlorophenols due to an excess incidence and mortality seen for certain cancers, including STS, in exposed workers.^37^ STS is also one of the few tumors specifically linked to dioxins, and 2,3,7,8-tetrachlorodibenzo-p-dioxin, considered the most potent dioxin, is classified as a group 1 carcinogen by the International Agency for Research on Cancer.^41^

The available clinicopathological characteristics of the present STS cohort stratified by sex revealed no significant differences apart from the tumor site, with the retroperitoneum more often involved in females, and the limbs or head and neck region in males. A possible explanation for this sex difference in primary sites might be that the limbs and the head and neck are most exposed to the abovementioned occupational mutagens.

Finally, concerning the direct costs of health care for STS in the 2 years after its diagnosis, the survival-weighted mean and median costs incurred did not differ to any statistically significant degree between the two sexes. The lack of sex differences in the cost of care during the 2 years after diagnosis and in the stage-adjusted survival may indicate that treatment did not differ; however, we found the likelihood of accessing the surgical procedure approach statistical difference, with lower odds in males. Further research is needed to confirm our results.

### Strengths and limitations

Although the population-based setting allows for homogeneous data collection, the database used here lacks detailed information on either potential environmental mutagens or host-related cancer risk factors. Other variables, such as comorbidities, that might be associated with sex differences in STS patients' survival or health care costs, were also unavailable.

## Conclusion

The present study showed a rising incidence of STS among males in the last three decades, but stable rates for females. Since the men and women in our sample shared much the same living environments, this difference might be explained by an occupational exposure to specific mutagens in jobs that are largely done by men.

## Data Availability Statement

The data supporting the findings of this study are held by the Veneto Epidemiological Registry and were used under license for the present work, but they are not publicly available. They are nonetheless available from M.Z. on reasonable request and subject to permission being obtained from the Veneto Epidemiological Registry (Veneto Regional Authority).
